# Laser-Cladding Cu-Cr-X Coating on Cu Alloy for Longer Service Life in Electrical Applications

**DOI:** 10.3390/ma18051103

**Published:** 2025-02-28

**Authors:** Xing Li, Lekang Lu, Jiashu Fang, Junjia Liang, Yesong Yang, Xiaojun Zhao, Sainan Liu, Lairong Xiao, Zhenyang Cai

**Affiliations:** 1 School of Materials Science and Engineering, Central South University, Changsha 410083, China; 213101027@csu.edu.cn (X.L.); lulekang2025@163.com (L.L.); jerryliangincsu@163.com (J.L.); 19973137008@163.com (Y.Y.); zhaoxj@csu.edu.cn (X.Z.); xiaolr@csu.edu.cn (L.X.); 2 Powder Metallurgy Research Institute, Central South University, Changsha 410083, China; 8204212428@csu.edu.cn; 3 Key Laboratory of Non-Ferrous Metal Materials Science and Engineering, Ministry of Education, Central South University, Changsha 410083, China; 4 School of Resource Processing and Bioengineering, Central South University, Changsha 410083, China; lsn@csu.edu.cn

**Keywords:** Cu-Cr-X coating, laser-cladding, wear resistance, service life

## Abstract

Advancements in electrical components have intensified the challenges for copper alloy wear resistance and high-temperature performance in electrical applications. The surface coating preparation of Cu alloys is crucial for enhancing their lifespan and promoting sustainable resource development. This study explored the microstructure and properties of Cu-Cr-X coatings (X = Mo/W, Al_2_O_3_/TiO_2_) on Cu alloy substrates via laser-cladding to improve wear resistance and hardness, vital for electrical component reliability and switching capacity. The process involved adjusting the power and reinforcing the phase particle size. The results showed hardness > 110 HV for all coatings (vs. 67.4 HV for the substrate). Cu-Cr-W achieved the highest hardness at 179 HV due to W dispersion and WCr precipitate reinforcement. It also maintained a stable CoF and the lowest wear rate (1.87 mg/km), with a fivefold wear resistance compared to the substrate alone. Cu-Cr-W excelled in lifespan extension and material loss reduction due to superior hardness, wear resistance, and conductivity.

## 1. Introduction

In recent decades, maintaining ecological balance has become a priority, fostering greater focus on natural resource conservation, green industries, and sustainable technologies. The United Nations, in its 2015 adoption of the “2030 Agenda for Sustainable Development Goals”, explicitly emphasizes the necessity of preventing the depletion of natural resources and pursuing greener industrial processes. Within this context, advancements in coating technology in the industrial sector [1,2] have been particularly beneficial, significantly extending material lifespan and contributing to broader sustainability goals.

Copper and its alloys exhibit exceptional electrical and thermal conductivity, making them ideal for optical cables and electronic components. Their applications are widespread, encompassing electrical engineering, mechanical systems, aerospace technology, and metallurgical equipment. Notably, copper alloys for electrical contacts are crucial in current conversion, commonly serving as the core in electrical switches for establishing and interrupting current flow. The performance of these materials is pivotal to the contact reliability and switching capabilities of switches. With advancements in technology within the field of electrical components, the copper alloys used in electrical applications are confronted with greater challenges in terms of wear resistance and high-temperature performance [3,4,5]. As a viable approach to achieving enhanced properties, the surface coating preparation of copper alloys has emerged as a promising method [6,7,8].

Laser-cladding technology represents a novel approach to surface coating technology, leveraging the energy provided by high-energy laser beams [9,10,11,12]. Through the irradiation and scanning of the coating layer using high-energy laser beams, selected coating materials are incorporated onto the surface of the melted workpiece utilizing varying powder-feeding methods. Specifically, Cu-Cr-based laser-cladding coating materials have been proven effective in enhancing the performance of electrical contact materials [13]. Copper chromium precipitation hardening (solution treatment, quenching, and aging) can achieve high strength and good conductivity. Anoop et al. [14] developed a method of Cu-3.4Cr-0.6Nb (at%) coating by laser in situ generation, and the synthesized alloy had a conductivity of 68% IACS and a hardness of 146 HV at room temperature. Within the academic community, two distinct technical routes have been explored: one involves the doping of Cu-Cr with wear-resistant metals, while the other involves doping with ceramics. This study has prepared two different systems of coatings: Cu-Cr doped with metals and Cu-Cr doped with ceramics [15]. Composite coatings composed of hard metals and ceramic materials possess favorable electrical conductivity, while the reinforcement of hard metals and ceramics contributes to enhancing the wear resistance of copper alloys. For instance, oxide materials such as Al_2_O_3_ [16,17] and TiO_2_ [17,18], along with hard metals like W [19] and Mo [20], have been found to be beneficial in this regard.

Therefore, the purpose of this study was to prepare Cu-Cr-Mo/W and Cu-Cr-Al_2_O_3_/TiO_2_ coatings through laser-cladding technology, regulating the power of the laser-cladding equipment and the size of the strengthening-phase particles. The microstructure and performance of the coating were studied, and the formation mechanism of the microstructure was analyzed.

## 2. Materials and Methods

Cu alloy (composition: Cr: 0.2–1.2%, Zr: 0.03–0.3%, Cu: balance, others: maximum 0.2%) served as the substrate for this study. The copper alloy substrate was precisely cut into specimens of dimensions 60 × 30 × 8 mm^3^ using electric-spark wire-cutting technology. Prior to further processing, the substrate surfaces were thoroughly cleaned by removing oxides and grease using 600-grit sandpaper (Shanghai Difeng New Materials Co., Ltd., Shanghai, China). Following sandblasting, the substrate was subjected to ultrasonic cleaning in an alcohol solution for 5 min to ensure the complete removal of contaminants, followed by drying.

Cu, Cr, Mo, W, Al_2_O_3_, and TiO_2_ raw powders were processed into a slurry using the ball-milling method, with deionized water as the dispersant. The powders measured in Step 1 were added, and a polyvinyl alcohol (PVA) binder was used to prepare the slurry for spray-drying. The binder content was 0.6–0.8 wt%, the dispersant content was 49.2–49.4 wt%, and the solid content was 50%. The slurry was mixed in a planetary ball mill with a corundum grinding ball at a ball-to-powder ratio of 1:1 for 4 h. The diameters of the grinding balls were classified into three specifications: large grinding balls with a diameter of 9 mm, medium grinding balls with a diameter of 5 mm, and small grinding balls with a diameter of 3 mm. The ratio of large, medium, and small grinding balls was 3:5:2 (wt%).

Subsequently, spherical powder materials suitable for laser-cladding were prepared by spray-drying, with an inlet temperature of 280 °C to 320 °C, an outlet temperature of 70 °C to 90 °C, and an atomizer rotation frequency of 35 Hz. The granulated powders were sintered at 300 –400 °C for 4 h to remove the binder, resulting in powders for laser-cladding.

**Figure 1:**
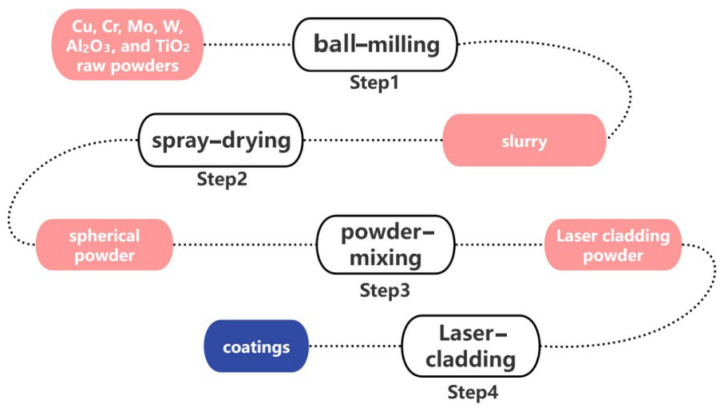
Schematic diagram of experimental section.

The ratio of Cu to Cr (vol%) in the four sets of laser-cladding Cu-Cr-X coating powders was 5:4, and the ratio of X (vol%) was 5%. The mixed powders for the four sets of experiments were composed of spherical copper powder, chromium powder, and X powder (Mo, W, Al_2_O_3_, and TiO_2_) obtained through spray-granulation. The mixed powders were blended using a three-dimensional powder mixer (Pengdong Three-Dimensional Powder Mixer SYH-5, Changzhou, China) at a speed of 150 rpm for 6 h. Next, we used synchronous powder-feeding laser-cladding equipment to prepare coatings on the surface of the CuCrZr alloy substrate. The entire coating preparation process is shown in Figure 1.

The laser-cladding equipment utilized in this study was an HC-6000-70R-60A fiber laser manufactured by Nanjing Zhongke Yuchen Laser Technology Co., Ltd. (Nanjing, China). The specific laser-cladding parameters (Optimization parameters obtained from preliminary experiments) are shown in Table 1.

Prior to conductivity measurements, the surface coating of the copper alloy obtained through coaxial powder-feeding and laser-cladding was polished sequentially with 400-mesh, 800-mesh, 1200-mesh, 1500-mesh, and 2000-mesh sandpaper. Conductivity assessments were conducted using a eddy current conductivity meter (Sigma 2008A, Xiamen Tianyan Instrument Co., Ltd., Xiamen, China) at 120 kHz, with each sample undergoing at least five repeated measurements. Vickers microhardness was determined on a polished sectional surface using a Vickers hardness tester (200 HBVS-30, Laizhou Huayin Testing Instrument Co., Ltd., Laizhou, China), applying a load of 5 N for a dwell time of 15 s. Measurements were taken at least three times, spanning from the coating’s surface to the substrate. These measurements were performed at intervals of 50–200 μm from the coating’s surface to the substrate, with each interval measured at least three times.

To evaluate the coating’s friction and wear properties, specimens after grinding and polishing were used. An HT-1000 high-temperature friction and wear tester was employed in accordance with ASTM G99-04 [21]. During the experiments, the friction coefficients were automatically recorded by the testing device (RLT-2M, Lanzhou Zhongke Kaihua Technology Development Co., Ltd., Lanzhou, China). The weight of the samples was measured before and after wear using a balance with an error of less than 1 mg. The wear performance was evaluated based on the sample mass (mg/km) lost per kilometer of wear. To minimize errors, the experiment was repeated three times.

## 3. Results and Discussion

### 3.1. Microstructure

**Figure 2:**
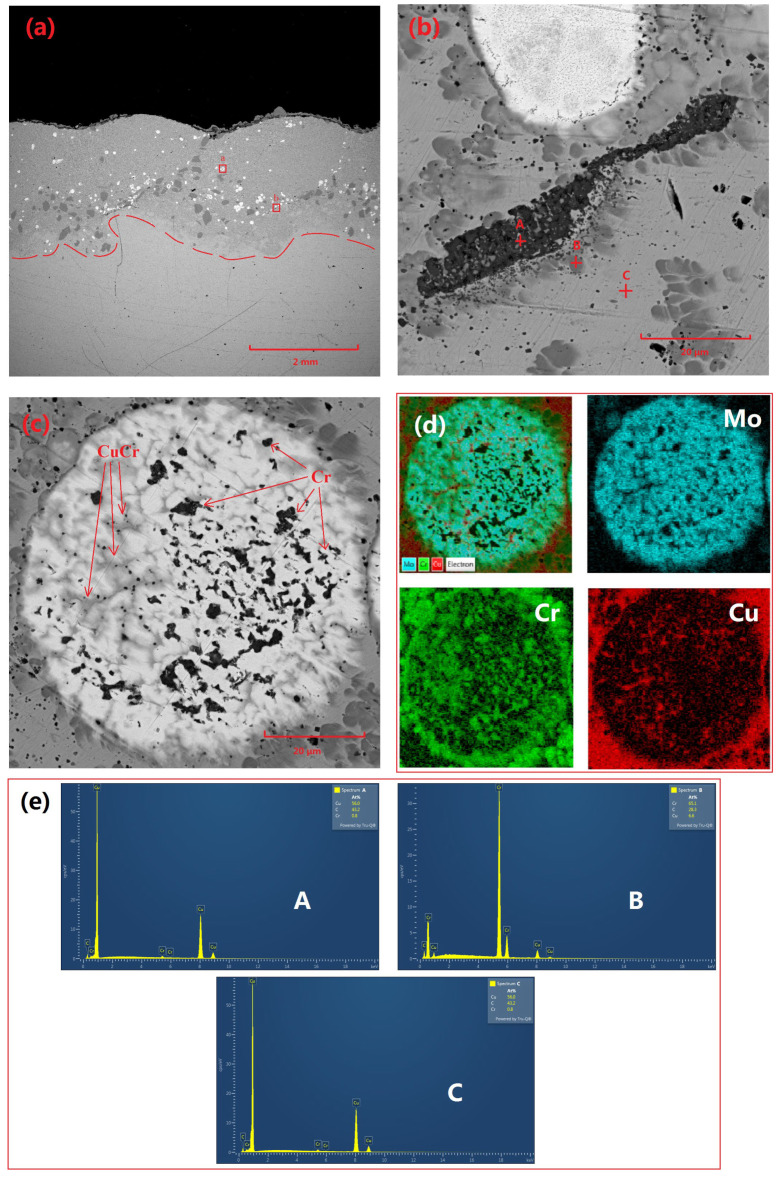
Microscopic morphology and elemental distribution of Cu-Cr-Mo coating: (**a**) macroscopic morphology of coating, (**b**) microscopic morphology of Cr-rich region (indicated by the b red box in (**a**)), (**c**) microscopic morphology of Mo particles (indicated by the a red box in (**a**)), (**d**) elemental distribution in (**c**), and (**e**) EDS spectra for points A, B, and C in (**b**).

Figure 2 demonstrates the microstructural characteristics and elemental distribution of the Cu-Cr-Mo coating. Specifically, Figure 2a presents the overall morphology of the coating, revealing a thickness of approximately 2 mm. The coating surface exhibits distinct wavy features, which are typical of laser-cladding surfaces. The coating interior primarily consists of Cu-based Cr precipitates, Cr-rich phases, and Mo particles. Among these, Mo particles are relatively dispersed, while Cr-rich regions primarily occur near the bottom of the coating adjacent to the substrate. This may be attributed to the faster cooling rate at the surface of the molten pool generated during laser-cladding, which does not allow sufficient time for Cr precipitates to grow and agglomerate. In contrast, the slower cooling rate near the substrate allows Cr precipitates to grow and agglomerate. Figure 2b shows partially unmelted residual Cr particles. Given that the diameter of the spray-agglomerated powder particles ranges from 50 to 80 μm, a few large-diameter Cr particles exhibit an unmelted state internally.

A comparison of Cu and Cr across different regions in Table 2 reveals that there is virtually no Cu element present in the residual particles. Additionally, due to the affinity of Cr for oxygen, there is a certain degree of oxidation present in Cr precipitates. A CuCr composite phase surrounds the Cr residual particles, which is a product of the surface melting of Cr residual particles and subsequent precipitation during cooling in adjacent locations. Figure 2c displays the morphology of incompletely melted molybdenum particles, characterized by high sphericity and a large number of pores within the particles. Elemental analysis indicated that these pores were filled with Cr and CuCr composite phases.

**Figure 3:**
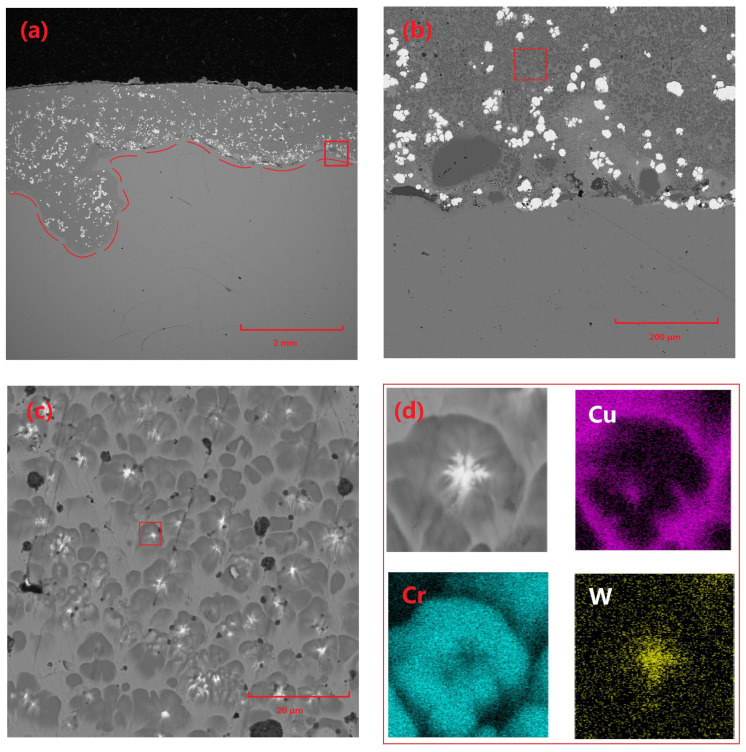
Microscopic morphology and elemental distribution of Cu-Cr-W coating: (**a**) macroscopic morphology of coating 2#, (**b**) morphology of the area indicated by the red box in (**a**), (**c**) morphology of the area indicated by the red box in (**b**), and (**d**) morphology and elemental distribution of the area indicated by the red box in (**c**).

Figure 3 presents the microstructural characteristics and elemental distribution of the Cu-Cr-W coating. Specifically, Figure 3a depicts the overall morphology of the coating, with a thickness of approximately 2 mm and a highly planar surface. The coating interior is primarily composed of Cu-based Cr precipitates and W particles, with a minimal volume of Cr-rich phases observed near the substrate edge. The W particles within the coating are relatively dispersed and mostly exhibit irregular block shapes. This is attributed to the high density of W, which results in the powder particles possessing significant inertia under the loading of high-speed gas flow during the cladding process, making them prone to disintegration under stress.

Figure 3c shows that the main structure of the coating consists of a Cu matrix, precipitated Cr structures, and a very small amount of Cr-rich phases. Figure 3c provides a detailed microscopic analysis of the fine structure of Cr precipitates within the coating, revealing that these precipitates generally contain significant heterogeneous components. Subsequent elemental analysis techniques confirm that these heterogeneous components are primarily W. This phenomenon can be attributed to several factors: Firstly, the refinement of W powder during ball-milling results in extremely small particle sizes, enabling them to absorb energy more efficiently and melt rapidly during laser-cladding. Subsequently, during the cooling and solidification of the molten pool, the high melting point of W causes it to precipitate first and undergo some coarsening. Following this, Cr utilizes the already precipitated W phases as nucleation cores for subsequent crystallization, ultimately forming a unique structure where Cr precipitates surround and encapsulate W particles.

Furthermore, the widely distributed W particles within the molten pool serve as effective nucleation sites, significantly promoting the precipitation and dispersion of Cr phases, thereby restricting the further growth of Cr precipitates. The prevalence of this nucleation mechanism provides a reasonable explanation for the rarity of Cr-rich regions within the coating as a whole, underscoring the uniformity and stability of the composite coating’s microstructure.

**Figure 4:**
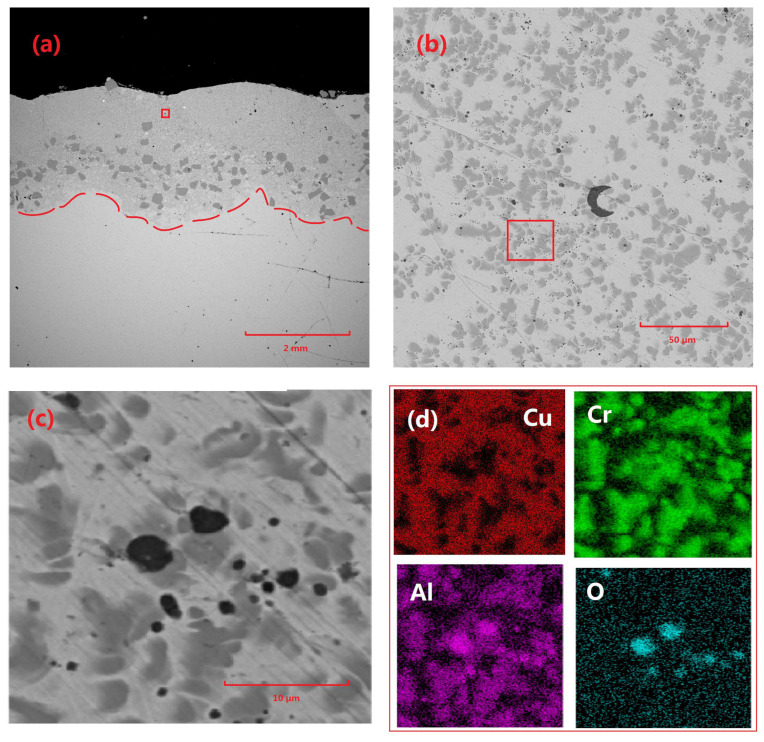
Microscopic morphology and elemental distribution of Cu-Cr-Al_2_O_3_ coating: (**a**) macroscopic morphology of coating 3#, (**b**) morphology of the area indicated by the red box in (**a**), (**c**) morphology of the area indicated by the red box in (**b**), and (**d**) elemental distribution of the area indicated by the red box in (**c**).

Figure 4 illustrates the microstructural characteristics and elemental distribution of the Cu-Cr-Al_2_O_3_ coating. Specifically, Figure 4a shows the overall morphology of the coating, with a thickness of approximately 2 mm and a wavy surface texture. The coating interior is primarily composed of Cu-based Cr precipitates and Cr-rich phases, which are predominantly concentrated in the lower portion of the coating. Further magnification reveals the presence of particles with diameters ranging from 2 to 3 μm to a maximum of approximately 10 μm within the coating. The elemental analysis confirmed that these particles were residual Al_2_O_3_. Additionally, a significant distribution of Al can be observed within the Cr precipitates.

The possible explanation for this phenomenon is that, while a small portion of Al_2_O_3_ particles remained unmelted and formed residual particles, the majority of them melted during the laser-cladding process and reacted with other components in the coating, particularly Cr, to form Al by deoxidation. During the precipitation process, the crystal structures of Al and Cr were both FCC (face-centered cubic), and their lattice parameters were relatively close, leading to the agglomeration and precipitation of CrAl.

**Figure 5:**
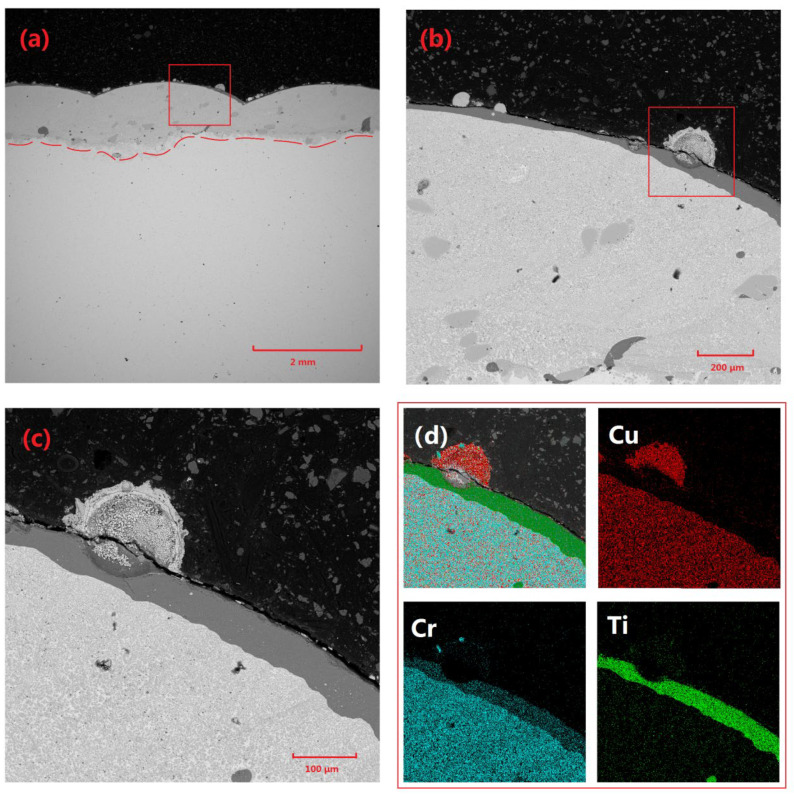
Microscopic morphology and elemental distribution of Cu-Cr-TiO_2_ coating: (**a**) macroscopic morphology of coating 4#, (**b**) morphology of the area indicated by the red box in (**a**), (**c**) morphology of the area indicated by the red box in (**b**), and (**d**) morphology and elemental distribution of the area indicated by the red box in (**c**).

Figure 5 demonstrates the microstructural characteristics and elemental distribution of the Cu-Cr-TiO_2_ coating. Specifically, Figure 5a shows the overall morphology of the coating, with a thickness of approximately 1.4 mm and a wavy surface texture. The coating interior is primarily composed of Cu-based Cr precipitates and Cr-rich phases. Further magnification reveals the presence of liquid-solidified structures that are completely heterogeneous to the substrate at the interface between the coating surface and the substrate. The elemental analysis identified this structure as metallic Ti. The possible reason is that during the laser-cladding process, TiO_2_ melted and reacted with other components to reduce and form metallic Ti. Since the density of metallic Ti is 4.5 g/cm^3^, which is much lower than that of Cu and Cr, Ti underwent upward circulation in the melt pool due to Stoke’s effect and floated to the surface of the coating. Additionally, the crystal structure of metallic Ti comprises an hexagonal α-phase and a cubic β-phase, whereas Cu and Cr in the coating were both face-centered cubic (FCC). Therefore, Ti did not precipitate and grow together with Cu and Cr, ultimately forming a Ti structure which floated on the surface of the coating.

### 3.2. Characterization

**Figure 6:**
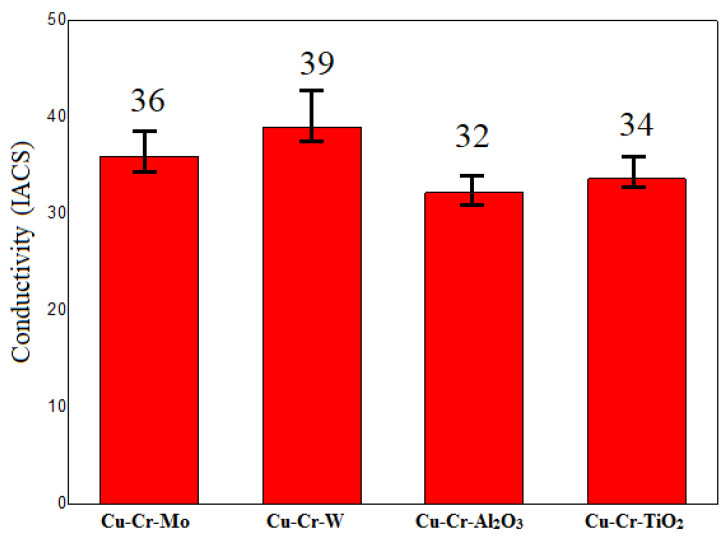
Coating electrical conductivity.

The electrical conductivity of the four coating groups is presented in Figure 6. It can be observed that the electrical conductivity of all four coating groups was greater than 30% IACS. Among them, the Cu-Cr-W coating exhibited the highest electrical conductivity, reaching up to 39% IACS. The primary reason for this was the immiscibility of W and Cu, having the least impact on the electrical conductivity of the coating. Additionally, the uniformly dispersed WCr composite precipitates prevented the formation and growth of Cr-rich phases, enhancing the continuity of Cu monometallics in the coating. This, in turn, reduced the influence of the second phase on the electrical conductivity of the coating.

**Figure 7:**
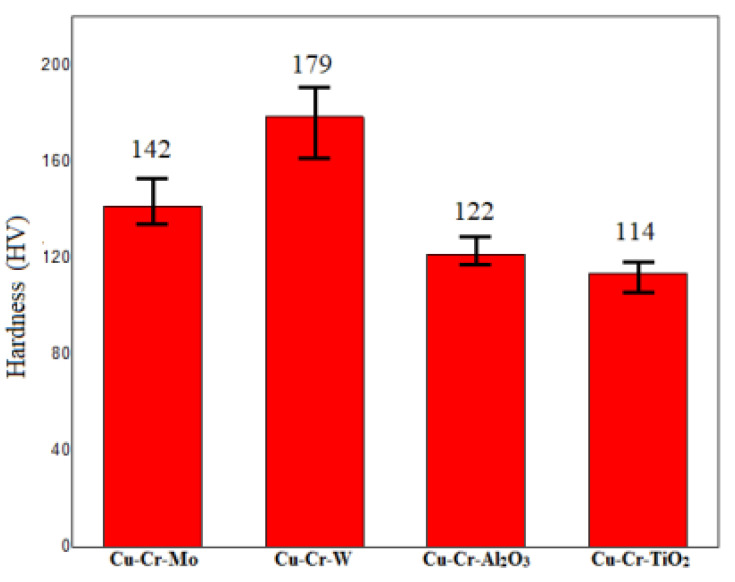
Coating hardness.

Figure 7 presents the hardness test results of four different coatings. The hardness values of all tested coatings were above 110 HV, significantly higher than the substrate material [15] (67.4 HV). Specifically, the Cu-Cr-W coating exhibited the highest hardness, reaching 179 HV. This may be attributed to the dispersion of hard metal W and the strengthening effect of the WCr composite’s dispersed precipitates on the coating. The two oxide-doped coatings exhibited a relatively lower hardness, which could be explained by the reduction in oxides during the laser-cladding process, forming metallic elements. Among them, Al was primarily distributed within the coating and formed AlCr intermetallic compounds, contributing to a certain degree of doping-strengthening effect. In contrast, Ti primarily floated on the surface of the coating, resulting in the lowest hardness.

**Figure 8:**
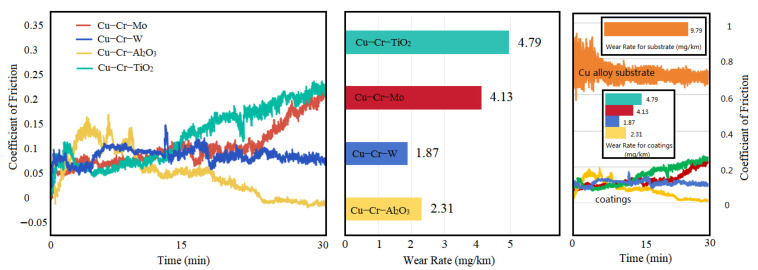
Coefficient of friction and wear rate (mg/km) of the coatings and substrate.

Figure 8 illustrates the variation in the coefficient of friction (CoF) and wear rate for four different coatings. The results indicate that, at the initial stage of the friction test, the CoF of three coatings increased due to Hertzian contact. After the sliding surfaces achieved stable mating, the CoF decreased and remained stable for a period. Subsequently, the CoF of Cu-Cr-Mo and Cu-Cr-TiO_2_ coatings underwent a significant increase, which could be attributed to the expansion of the friction surface and the softening of Cu within the coatings, leading to adhesive wear as wear progressed. In contrast, the CoF of the Cu-Cr-W coating remained relatively stable, while that of the Cu-Cr-Al_2_O_3_ coating decreased slightly. In terms of wear rate, the Cu-Cr-Mo and Cu-Cr-TiO_2_ coatings exhibited more severe wear conditions due to their higher CoF at later stages, both exceeding 4 mg/km. The Cu-Cr-W coating, benefiting from its lower CoF and the strengthening effect of dispersed hard phases and WCr composite precipitates, achieved the lowest wear rate of 1.87 mg/km. Although the Cu-Cr-Al_2_O_3_ coating had the lowest CoF, its microstructure was less hard than that of the Cu-Cr-W coating, resulting in a slightly higher wear rate. Simultaneously, a set of friction and wear tests were conducted on a copper alloy substrate under identical experimental conditions. The results indicate that the friction coefficient of the copper substrate ranged between 0.6 and 0.8, while the friction coefficients of all four coating groups were below 0.25. The significantly higher friction coefficient of the copper alloy substrate compared to the coatings was attributed to the relatively lower hardness and greater deformability of the copper alloy, leading to severe adhesive wear. The wear rate of the copper alloy substrate was 9.79 mg/km, approximately twice that of the coating with the highest wear rate (Cu-Cr-TiO_2_) and nearly five times that of the coating with the lowest wear rate (Cu-Cr-W). It is evident that these coatings could effectively reduce the friction and wear properties of the copper alloy substrate, enhancing component lifespan and decreasing the material loss rates.

**Figure 9:**
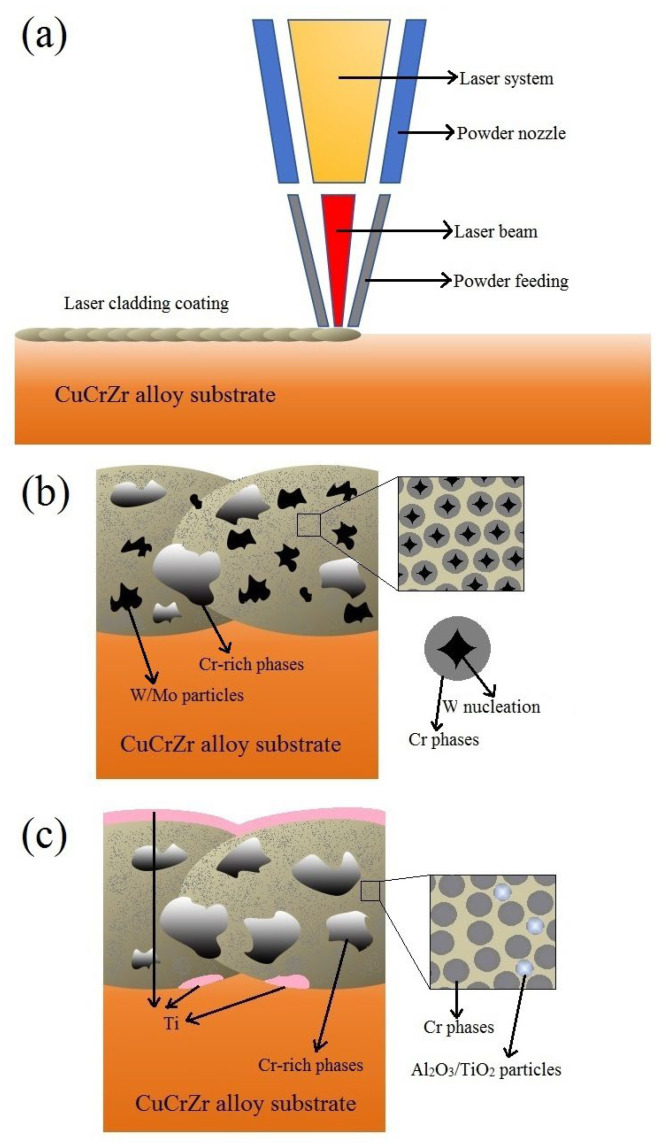
Schematic diagram of laser-cladding process (**a**); schematic illustration of the Cu-Cr-W/Mo coating (**b**); and schematic illustration of the Cu-Cr-Al_2_O_3_/TiO_2_ coating (**c**).

Based on the integration of several experimental sets, it can be observed that, during the laser-cladding process, the high-energy laser beam interacted with the coating material powder simultaneously delivered through a powder nozzle on the substrate surface, forming a molten pool and subsequently solidifying into a coating (Figure 9a). During this process, different powder raw materials, due to their distinct melting, cooling, and solidification mechanisms, resulted in varied coating microstructures. CuCr and hard metal powders, such as W in Cu-Cr-W coatings, were capable of more fully absorbing energy and rapidly melting. During the cooling and solidification phase of the molten pool, W precipitated first from the liquid phase and underwent a certain degree of coarsening. Subsequently, Cr utilized the already precipitated W phase as nucleation sites for subsequent crystallization and precipitation, ultimately forming a unique structure where Cr precipitates surrounded and encapsulated W particles (Figure 9b). Conversely, when CuCr was combined with oxide ceramics, such as in the Cu-Cr-Al_2_O_3_ coatings, the oxide interacted with the CuCr substrate at high laser temperatures to form lightweight simple metal substances that adhered to the coating surface, leading to an inhomogeneous structure which affected the uniformity of coatings and wear resistance (Figure 9c). Additionally, the dissolution of lightweight metals in the coating similarly reduced the coating’s electrical conductivity.

## 4. Conclusions

Spherical powders with good sphericity and high fluidity were prepared using ball-milling combined with spray-drying technology. These powders were mixed in specific proportions to form four types of coating raw powders: Cu-Cr-Mo, Cu-Cr-W, Cu-Cr-Al_2_O_3_, and Cu-Cr-TiO_2_. After laser-cladding, the resulting coatings exhibited a thickness ranging from 1.6 mm to 2 mm. The coatings were fully metallurgically bonded to the substrate and were dense and smooth.

All four coatings exceed 110 HV in hardness, significantly harder than the substrate (67.4 HV). Notably, Cu-Cr-W achieved the highest hardness at 179 HV due to W dispersion and WCr precipitate strengthening. Oxide-doped coatings exhibited a lower hardness due to oxide reduction. Initially, the coefficient of friction (CoF) increased due to Hertzian contact but stabilized. Cu-Cr-Mo and Cu-Cr-TiO_2_ CoF subsequently rose, potentially due to surface expansion and Cu softening, leading to higher wear rates exceeding 4 mg/km. In contrast, Cu-Cr-W maintained a stable CoF and achieved the lowest wear rate at 1.87 mg/km. Despite Cu-Cr-Al_2_O_3_′s lowest CoF, its microstructure’s softer hardness resulted in slightly higher wear compared to Cu-Cr-W. Simultaneously, the wear resistance performance indicators, such as the coefficient of friction and wear rate, of the four coating groups were significantly superior to those of the copper alloy substrate, demonstrating the advantages of the coatings in enhancing component lifespan and reducing material loss.

All four coating groups exhibited electrical conductivity > 30% IACS, with Cu-Cr-W demonstrating the highest at 39% IACS. This was attributed to the immiscibility of W and Cu, minimally affecting conductivity, and uniform WCr precipitate dispersion preventing Cr-rich phase formation, enhancing Cu continuity and reducing second-phase impact on conductivity.

Distinct powder raw materials yield varied coating microstructures due to different melting, cooling, and solidification processes. CuCr and hard metal powders (e.g., Cu-Cr-W) fully absorb energy and melt rapidly. In our study, during cooling, W precipitated and coarsened first, with Cr subsequently crystallizing around the W particles. This structure endowed the Cu-Cr-W coating with the highest hardness, lowest wear rate, and best electrical conductivity among the four coating groups, indicating promising applications. In contrast, CuCr combined with oxide ceramics (e.g., Cu-Cr-Al_2_O_3_) formed lightweight metal substances at high laser temperatures, causing inhomogeneous structures which affected uniformity and wear resistance. Lightweight metal dissolution also reduced electrical conductivity.

## Figures and Tables

**Table 1:** Laser-cladding process parameters.

Parameter Category	Parameter Value
Power	3 kW
Wavelength	1080 nm
Laser Speed	0.9 m/min
Powder Feed Rate	1.5 rpm
Step Distance	2.1 mm
Overlap Ratio	30%
Carrier Gas (Helium)	15 L/min

**Table 2:** Elemental distribution at selected points in Figure 2b.

Element	Cu	Cr	O
A point (wt%)	1.39	55.71	32.76
B point (wt%)	10.19	81.61	-
C point (wt%)	86.37	1.02	-

## Data Availability

The original contributions presented in this study are included in the article. Further inquiries can be directed to the corresponding author.
